# A Randomized Controlled Trial to Compare the Safety and Efficacy of Tadalafil and Tamsulosin in Relieving Double J Stent Related Symptoms

**DOI:** 10.1155/2015/592175

**Published:** 2015-12-14

**Authors:** Satinder Pal Aggarwal, Shivam Priyadarshi, Vinay Tomar, S. S. Yadav, Goto Gangkak, Nachiket Vyas, Neeraj Agarwal, Ujwal Kumar

**Affiliations:** SMS Medical College and Hospital, Jaipur 302004, India

## Abstract

*Objectives*. To evaluate the safety and efficacy of Tadalafil and Tamsulosin in treating Double J stent related symptoms.* Methods*. In a prospective study, 161 patients with DJ related symptoms were randomized into 3 groups: Group A patients (54), Group B patients (53), and Group C patients (54). They were given Tadalafil, Tamsulosin, and placebo, respectively, at 1st week till removal of DJ stent at 3rd week. All patients completed Ureteral Stent Symptom Questionnaire (USSQ) at 1st week and at 3rd week. The statistical significant difference among groups was determined by the *t*-test, Kruskal-Wallis test and multivariate analysis were used to assess association of the variables within the three groups, and the level of significance was set at *P* < 0.05.* Results*. Tadalafil and Tamsulosin were comparable in relieving urinary symptoms, general health, and work performance (OR = 0.65, 1.8, and 0.92). But Tadalafil was more effective in relieving body pain, sexual problems, and additional problems than Tamsulosin (OR = 5.95, 19.25, and 2.69) and was statistically significant as *P* < 0.05.* Conclusion*. Tadalafil was as effective as Tamsulosin in relieving urinary symptom but more effective in relieving sexual symptoms and body pain.

## 1. Introduction

Since its first introduction by Zimskind et al. in 1967, endoscopic stent placement has become an indispensable part of urology [1]. Early era was plagued with frequent stent migration and expulsion. Development of the Double J (DJ) and pigtail stents by Finney and Hepperlen (1978) solved these problems, making ureteral stenting a routine urological procedure [2]. It is employed for relief of ureteral obstruction and ureteral injury and as a ureteral splint in various open, laparoscopic, and endourological procedures.

Despite its usefulness, DJ stent leads to morbid lower urinary tract symptoms (LUTS) such as frequency (50–60%), urgency (57–60%), dysuria (40%), flank pain (19–32%), suprapubic pain (30%), and hematuria (25%), affecting quality of life in approximately 80% of patients [3–5]. More than 80% of patients experience stent related pain affecting daily activities, 32% report sexual dysfunction, and 58% report reduced work capacity [5]. Joshi et al. had developed a validated self-administered Ureteral Stent Symptom Questionnaire (USSQ), for evaluating stent related symptoms in the clinical and research settings [6]. These symptoms are managed by alpha blockers, anticholinergics, and analgesics [7–18]. Tamsulosin, a selective *α*-1a/1d blocker, inhibits contraction of the smooth muscles in distal ureter, bladder trigone, and neck, relieving LUTS and flank pain.

PDE-5 receptors are present over lower ureter, trigone, and bladder neck. The role of Tadalafil, PDE-5 inhibitor, in sexual dysfunction, relieving bladder outlet obstruction related lower urinary tract obstruction (LUTS) and lower ureteric stone expulsion, is well studied. The purpose of this study was to evaluate and compare the efficacy and safety of Tadalafil and Tamsulosin.

## 2. Materials and Methods

This prospective placebo controlled double blind randomized study was conducted among 220 patients (154 men and 66 women) who underwent DJ stenting after uneventful endourological surgeries by a single surgeon from February 2014 to March 2015. The study protocol was approved by the institutional ethics committee and all patients enrolled in this study gave written informed consent.

All patients (aged 18 to 50 years) undergoing unilateral percutaneous nephrolithotomy (PCNL) or ureteroscopic lithotripsy URSL (bilateral normally excreting kidneys) with DJ stenting were evaluated for enrollment in the study. Patients with age less than 18 years and more than 50 years, patients taking nitrate drugs, patients with postoperative residual stone fragments, pregnant women, and patients with bilateral stents, long-term stenting (on regular change), bladder/prostate pathology (leading to irritative bladder symptoms: prostatomegaly, prostatitis, carcinoma prostate, overactive bladder, and neurogenic bladder), history of lower urinary tract surgery, and chronic use of selective alpha-1 blocker and/or anticholinergic agents were excluded from study.

URSL was done with 6.5/8.5 Fr ureteroscope (Wolf) and laser lithotripsy (holmium). PCNL was done using 26 Fr nephroscope (Wolf) and pneumatic lithotripter. The decision for stent placement was taken by operating urologist, depending upon large stone burden, mucosal trauma, and need for ureteral dilation to access tight ureters. 6 Fr and 26 cm long DJ stent composed of polyurethane material was put under fluoroscopy guidance. Postoperative X-ray KUB was done in all patients to rule out residual stone fragment. On day of surgery, injection of ceftazidime 1 gm i/v was given prophylactically to all patients. Foleys catheter was removed on 1st post-op day in both PCNL and URSL patients. Nephrostomy tubes were removed on 2nd post-op day in PCNL patients. Tab levofloxacin 500 mg OD was given for 7 days postoperatively, as per our institutional protocol. Patients were informed about DJ related symptoms and were given USSQ at discharge. They were asked to come after 1 week with completed questionnaire, if they experience symptoms. Scoring at 1st week was done to see the magnitude of DJ related symptoms.

**Figure 1:**
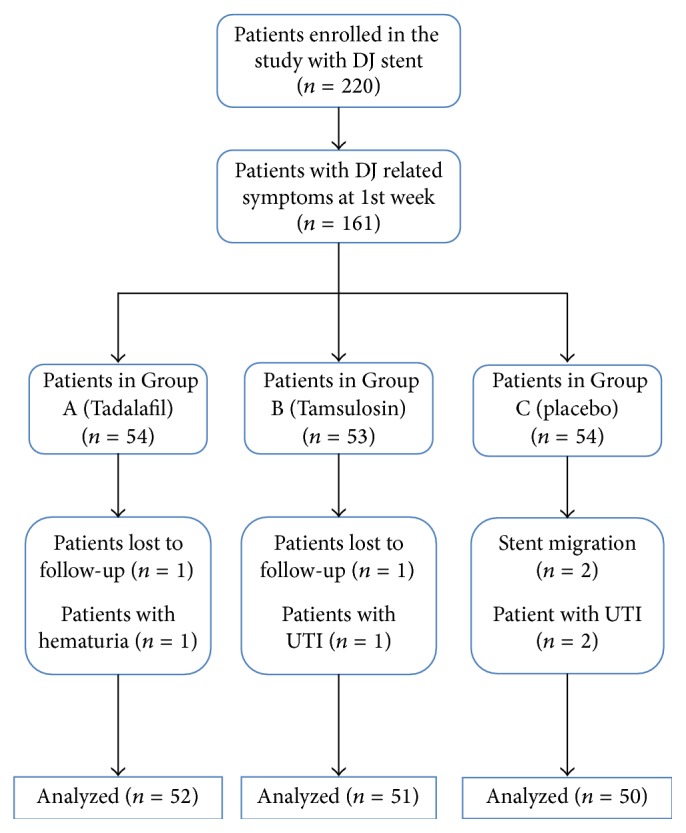
Study design.

After applying inclusion and exclusion criteria, 161 patients reported DJ related symptoms at 1st week and they were randomized into 3 groups (A, B, and C) in a ratio of 1 : 1 : 1 by computer generated module. Group A (54 patients) were put on Tab Tadalafil 5 mg OD, Group B (53 patients) were put on Tab Tamsulosin 0.4 mg OD, and Group C (54 patients) were put on placebo OD. Patients were advised to take analgesics (diclofenac) as per need. Tb Tadalafil 5 mg, Tb Tamsulosin 0.4 mg, and sugar coated placebo tablets were put in 3 identical bottles. Double blinding was done to minimize bias. Nursing staff gave the drug bottle to patient by chit method. All patients were informed about side effects of drugs. They were given USSQ and were asked to come with completed USSQ at 3rd week, before removal of DJ stent. Out of the 158 patients, 2 patients were lost to follow-up, 3 had urinary tract infection (UTI), 1 had hematuria (so early removal of stent), and 2 had stent migration. So data of these 8 patients were not analyzed (Figure 1). Analgesic requirement and side effects of drug during study period in each group were noted.

Data so collected was tabulated in an excel sheet, under the guidance of statistician. Data was analyzed using IBM SPSS Statistics Windows, Version 20.0 (Armonk, NY: IBM Corp.) for the generation of descriptive and inferential statistics. The statistical significant difference among groups was determined by the *t*-test, Kruskal-Wallis test and multivariate analysis were used to assess association of the variables within the three groups, and the level of significance was set at *P* < 0.05.

## 3. Results

Out of 220 patients, 161 patients complained of DJ related symptoms (73.2%). Mean age, male to female ratio, average height, and procedures performed were uniform in all 3 groups (Table 1).

Both Tadalafil and Tamsulosin led to significant decrease in urinary symptoms, body pain, sexual health, general health, and work performance scores at 3rd week as compared to 1st week score. Placebo does not lead to improvement in symptom score over study period. Also analgesic requirement was significantly less in Tadalafil group as compared to both Tamsulosin and placebo group (Table 2).

For comparing efficacy of Tadalafil with Tamsulosin, mean decrease in symptom score within each group was analyzed using Kruskal-Wallis test (Table 3). Decrease in urinary symptoms, work performance and additional problems were similar in Tadalafil and Tamsulosin group. Improvement in body pain, sexual health, and general health was significantly more in Tadalafil group than both Tamsulosin and placebo group.

When multivariate analysis was applied to assess mean symptom score difference of 1st and 3rd week among three groups, both Tadalafil and Tamsulosin are comparable in relieving urinary symptoms, general health, and work performance (OR = 0.65, 1.8, and 0.92) Table 4. But Tadalafil is more effective in relieving body pain, sexual problems, and additional problems than Tamsulosin (OR = 5.95, 19.25, and 2.69) and was statistically significant as *P* < 0.05. Side effects of Tadalafil and Tamsulosin were minimal. No patient left study due to side effects of drugs.

## 4. Discussion

DJ stenting is an integral part of today's urology practice. DJ stenting leads to LUTS in 80% of patients, leading to reduced health related quality of life (HrQOL). DJ related symptoms include frequency, urgency, dysuria, hematuria, flank pain, suprapubic pain, and sexual dysfunction [3–5]. Our understanding of pathophysiology of these symptoms is lacking but improving. Bladder mucosal irritation due to contact by the distal curl of the stent, ureteral smooth muscle spasm, and reflux of urine resulting in flank pain are the proposed mechanisms [4]. The USSQ evaluates stent related symptoms in six domains—urinary symptoms, body pain, general health, work performance, sexual performance, and other problems. The urinary symptoms domain has 11 questions. The body pain domain has pain experience, visual analog scale, and six questions. The general health, work performance, and sexual performance domains have six, seven, and four questions, respectively. Each question has 4 to 7 scores. Scores from each question are added to give total score, with higher score indicating more bothersome symptoms [6].

Management of DJ related symptoms is still improving with better understanding of pathophysiology of symptoms. Management is based on preventive and pharmaceutical methods. Preventive strategies include avoiding DJ stenting in uncomplicated cases, appropriate stent length as per patient height, proper positioning, drug eluting stents, and patient counseling regarding symptoms [19–25]. Since the inception of DJ stenting, the quest is still going on for improving DJ design and material to decrease DJ related morbidity. Various modifications in DJ stent design and material have led to reduction in DJ related symptoms [26]. Rane et al. advocated use of appropriate sized DJ, so that bladder curl does not cross the midline to minimize irritation of trigone and thus symptoms [27].

Alpha blockers, anticholinergics, or their combination is prescribed empirically. Alpha blockers result in a significant reduction in the peak contraction pressure, leading to ureteral dilation [28]. Thus, alpha blockers by decreasing muscle spasm and intrarenal urinary reflux may explain the ability to relieve flank pain. Irritative symptoms (frequency, dysuria, and urgency) may improve because of the alpha receptors blockage at the bladder trigone. Deliveliotis et al. were the first to demonstrate that Alfuzosin relieved the stent related symptoms, pain and improved sexual and general health [7]. Beddingfield et al. also concluded in their study that Alfuzosin 10 mg daily improved frequency and flank pain [8]. Similarly, Wang et al. reported that Tamsulosin improved urinary symptoms and flank pain during voiding [9]. More various trials have confirmed the efficacy of alpha blockers in relieving stent related symptoms, making alpha blockers the most commonly prescribed agents [10–13]. Similarly in our study, Tamsulosin was effective in relieving urinary symptoms, body pain, general health, and work performance as compared to placebo. Improvement in general health and work performance can be explained by decreased urinary and body pain symptom score.

Bladder coil of stent may irritate trigone and lead to OAB like symptoms. This hypothesis leads to use of anticholinergics in relieving DJ related symptoms. Lee et al. studied the role of solifenacin in placebo controlled randomized study and found it to be effective [14]. Similarly Park et al. reported efficacy of tolterodine in relieving stent related LUTS in their prospective randomized controlled study [15]. Assuming that both alpha and cholinergic receptors have a role to play in genesis of DJ stent related symptoms, studies have been done comparing combination with monotherapy, proving combination to be better than monotherapy [16–18].

PDE-5 inhibitors are rapidly expanding their therapeutic indications. They are FDA approved for managing erectile dysfunction, prostatomegaly related bladder outlet obstruction, and pulmonary hypertension. They are studied in various randomized trials for medical expulsion therapy for lower ureteric stone expulsion therapy. Tadalafil also relaxes ureter by blocking PDE-5 receptors present on lower ureter, thus reducing spasm and reflux. PDE-5 receptors are also present on bladder trigone and neck; thus by blocking these receptors irritative symptoms might be taken care of. Hajebrahimi et al. conducted a placebo controlled randomized trial to evaluate role of Tadalafil in relieving stent related symptoms. In their study, Tadalafil improved stent associated urinary symptoms, body pain, and sexual matter [29]. We conducted a randomized trial comparing three groups Tadalafil versus Tamsulosin versus placebo in relieving stent related symptoms unlike only couple of studies done in the past (one of which was pilot study and others involve only two groups, i.e., placebo and Tadalafil). So, our study in addition gives comparison in relieving stent related symptoms between Tadalafil and most commonly used alpha blocker Tamsulosin. In our study, Tadalafil was effective in relieving urinary symptoms, body pain, sexual health, general health, and work performance as compared to placebo. In comparison to Tamsulosin, Tadalafil is equally effective in relieving urinary symptoms, general health, and work performance but more effective in relieving body pain, sexual problems, and additional problems. Similar relief in urinary symptoms may be due to similar action of ureteral and trigonal smooth muscle. Increased sexual symptom improvement can be due to resolution of erectile dysfunction and decrease in body pain.

Ours is a single center study; DJ stent was not height specific, use of only single stent design and material which forms limitations of the study. Though the groups were randomized, the effect of same length DJ stent should not have any bearing on the difference in results under each group. Moreover, the average height of patients is also same in all the three groups. To validate the results of our study further large multicentric studies are required.

## 5. Conclusion

Tadalafil is safe and effective in relieving DJ related symptoms. Tadalafil is as effective as Tamsulosin in relieving urinary symptom but more effective in relieving sexual symptoms and body pain. Therefore Tadalafil could be recommended as preferred drug in sexually active patients undergoing DJ stenting.

## Figures and Tables

**Table 1:** Basic parameters of patients.

Variables	Group A	Group B	Group C
	Tadalafil	Tamsulosin	Placebo
Patients (*n*)	52	51	50
Mean age (years)	32.2	34.3	33.4
Sex (M : F)	37 : 15	35 : 16	38 : 12
Average height	5′6′′	5′7′′	5′6′′

Procedures			
PCNL	33	32	33
URSL	19	19	17

**Table 2:** Ureteral stent symptom score at 1st week and 3rd week in three groups.

	Group A (Tadalafil)		*t*-test	Group B (Tamsulosin)		*t*-test	Group C (placebo)		*t*-test
	1st week	3rd week		1st week	3rd week		1st week	3rd week	
Urinary symptoms	40 (12–50)	20.1 (4–24)	**<0.0001**	42.3 (10–51)	21.8 (4.2–28)	**<0.0001**	43 (15–51)	42 (16–49)	0.12
Body pain	20 (4–25)	7.1 (3–14)	**<0.0001**	17 (3–22)	13.4 (3.9–19)	**0.02**	18.8 (12–22)	17 (11–20)	0.06
Sexual health	8 (2–10)	2.9 (1–5)	**<0.0001**	7 (2.3–11)	5.9 (1.6–9)	**0.03**	9 (6–10)	8.2 (4–9)	0.08
General health	23 (13–27)	19.2 (9–22)	**<0.0001**	22 (14–28)	19.6 (7–21)	**<0.0001**	25.6 (11–27)	24.9 (13–25)	0.27
Work performance	14 (4–15)	12.2 (4–14)	**0.03**	12 (3.3–15)	10 (4–13)	**0.02**	15 (12–15)	14 (11–15)	0.27
Additional problems	13 (12–18)	12.4 (3–14)	0.32	12 (3.5–20)	11.8 (3–17)	0.21	16.6 (7–18)	16.3 (8–18)	0.51
Analgesic used Diclofenac (mg)	600^A^			1250^B^			2200^C^		<0.0001^*∗*^

^*∗*^Kruskal-Wallis test; values in the column with different letters indicate significant differences at *P* < 0.05.

**Table 3:** Comparison of mean difference of ureteral stent symptom score at 1st week and 3rd week in three groups.

Characteristics	Mean difference at 1st & 3rd week of three groups			*P* value
	Group A (Tadalafil)	Group B (Tamsulosin)	Group C (placebo)	
Urinary symptoms	19.9^A^	20.5^A^	1^C^	<0.0001
Body pain	12.9^A^	3.6^B^	1.8^C^	<0.0001
Sexual health	5.1^A^	1.1^B^	0.8^B^	<0.0001
General health	3.8^A^	2.4^B^	0.7^C^	<0.0001
Work performance	1.8^A^	2^A^	1^B^	<0.0001
Additional problems	0.6^A^	0.2^A^	0.3^A^	0.21

^*∗*^Kruskal-Wallis test; values in the column with different letters indicate significant differences at *P* < 0.05.

**Table 4:** Multiple regression analysis of mean ureteral stent symptom score at 1st week and 3rd week in three groups.

Variables	Group A Tadalafil		Group B Tamsulosin				Group C Placebo		
	*N*		*N*	OR (CI)	*P* value		*N*	OR (CI)	*P* value
Urinary symptoms >20	24		29	0.65 (0.30–1.42)	0.28		0	86.82 (5.09–991.42)	<0.0001
Body pain >10	23		6	5.95 (2.16–16.37)	0.0006		1	40.25 (5.15–314.32)	<0.0001
Sexual health >5	22		2	19.25 (4.21–88.03)	<0.0001		1	35.93 (4.60–280.50)	<0.0001
General health >3	31		22	1.8 (0.86–4.13)	0.12		2	35.43 (7.76–161.84)	<0.0001
Work performance >2	27		27	0.96 (0.44–2.08)	0.92		13	3.07 (1.34–7.08)	0.008
Additional problems >1	19		9	2.69 (1.08–6.71)	0.03		8	3.0227 (1.18–7.77)	0.02
